# Continuous daylight in the high-Arctic summer supports high plankton respiration rates compared to those supported in the dark

**DOI:** 10.1038/s41598-017-01203-7

**Published:** 2017-04-28

**Authors:** Elena Mesa, Antonio Delgado-Huertas, Paloma Carrillo-de-Albornoz, Lara S. García-Corral, Marina Sanz-Martín, Paul Wassmann, Marit Reigstad, Mikael Sejr, Tage Dalsgaard, Carlos M. Duarte

**Affiliations:** 1 Instituto Andaluz de Ciencias de la Tierra, CSIC-UGR, Avda. de las Palmeras 4, 18100 Armilla, Spain; 2 King Abdullah University of Science and Technology (KAUST), Red Sea Research Center (RSRC), Thuwal, 23955-6900 Saudi Arabia; 3 Instituto Mediterráneo de Estudios Avanzados (IMEDEA), CSIC-UiB, Miquel Marqués 21, 07190 Esporles, Spain; 4 Facultat de Geologia, Universitat de Barcelona, Barcelona, Spain; 5 Institute of Arctic and Marine Biology, UiT The Arctic University of Norway, N-9037 Tromsø, Norway; 6 Arctic Research Centre, Department of Bioscience, Aarhus University, Aarhus, Denmark

## Abstract

Plankton respiration rate is a major component of global CO_2_ production and is forecasted to increase rapidly in the Arctic with warming. Yet, existing assessments in the Arctic evaluated plankton respiration in the dark. Evidence that plankton respiration may be stimulated in the light is particularly relevant for the high Arctic where plankton communities experience continuous daylight in spring and summer. Here we demonstrate that plankton community respiration evaluated under the continuous daylight conditions present *in situ*, tends to be higher than that evaluated in the dark. The ratio between community respiration measured in the light (R_light_) and in the dark (R_dark_) increased as the 2/3 power of R_light_ so that the R_light_:R_dark_ ratio increased from an average value of 1.37 at the median R_light_ measured here (3.62 µmol O_2_ L^−1^ d^−1^) to an average value of 17.56 at the highest R_light_ measured here (15.8 µmol O_2_ L^−1^ d^−1^). The role of respiratory processes as a source of CO_2_ in the Arctic has, therefore, been underestimated and is far more important than previously believed, particularly in the late spring, with 24 h photoperiods, when community respiration rates are highest.

## Introduction

Community respiration is the process responsible for the degradation of organic matter by organisms to extract energy to support biological processes in the ecosystem and provides, therefore, an integrated assessment of the energy requirements of the ecosystem^1^. Oceanic respiration, estimated to release 66 Gt C year^−1^ globally, is one of the main elements of the carbon flux in the biosphere^2^, but remains the least constrained term in most models of metabolism, gas exchange and carbon mass balance in the ocean^3, 4^.

Our understanding of the respiration of plankton communities is also limited by the fact that most respiration rates have been evaluated using bulk oxygen consumption rates evaluated in the dark, thereby assuming respiration in the dark to be equivalent to that in the light^4^. However, published reports suggest that respiration in the light might be higher than that in the dark^5–7^, so current estimates of community respiration of plankton communities may be underestimated.

The severity of the bias involved in the assumption that community respiration in the dark equals that in the light involved in most estimates of plankton community respiration, depends on the photoperiod the community experiences. This shows the broadest range in the high Arctic, where there is an extended period of darkness in fall and winter, where darkness prevails, and an extended period of continuous daylight in spring and summer, when any differences between respiration in the dark and that in the light will have the highest impact on the estimates. The robust assessment of community respiration in the Arctic is particularly important, as community respiration has been predicted to rise with future Arctic warming^8–10^. Yet, the bias introduced by the assumption that community respiration in the dark equals that in the light in the Arctic summer has not yet been assessed. Here we evaluate plankton community respiration rates in the photic zone of the Arctic ocean along several cruises conducted in the spring/summer period in the European Sector of the Arctic Ocean, during 2012, 2013 and 2014. In particular, we test the null hypothesis that community respiration rate in the dark equals that in the light. We did so by calculating respiration rate using oxygen consumption in the dark, evaluated by high-precision Winkler titration, and estimating community respiration in the light as the difference between gross primary production (GPP^18^O), evaluated with the ^18^O method and net community production (NCP), evaluated from bulk oxygen mass balance, of communities incubated under ambient incoming irradiance.

## Results

**Figure 1:**
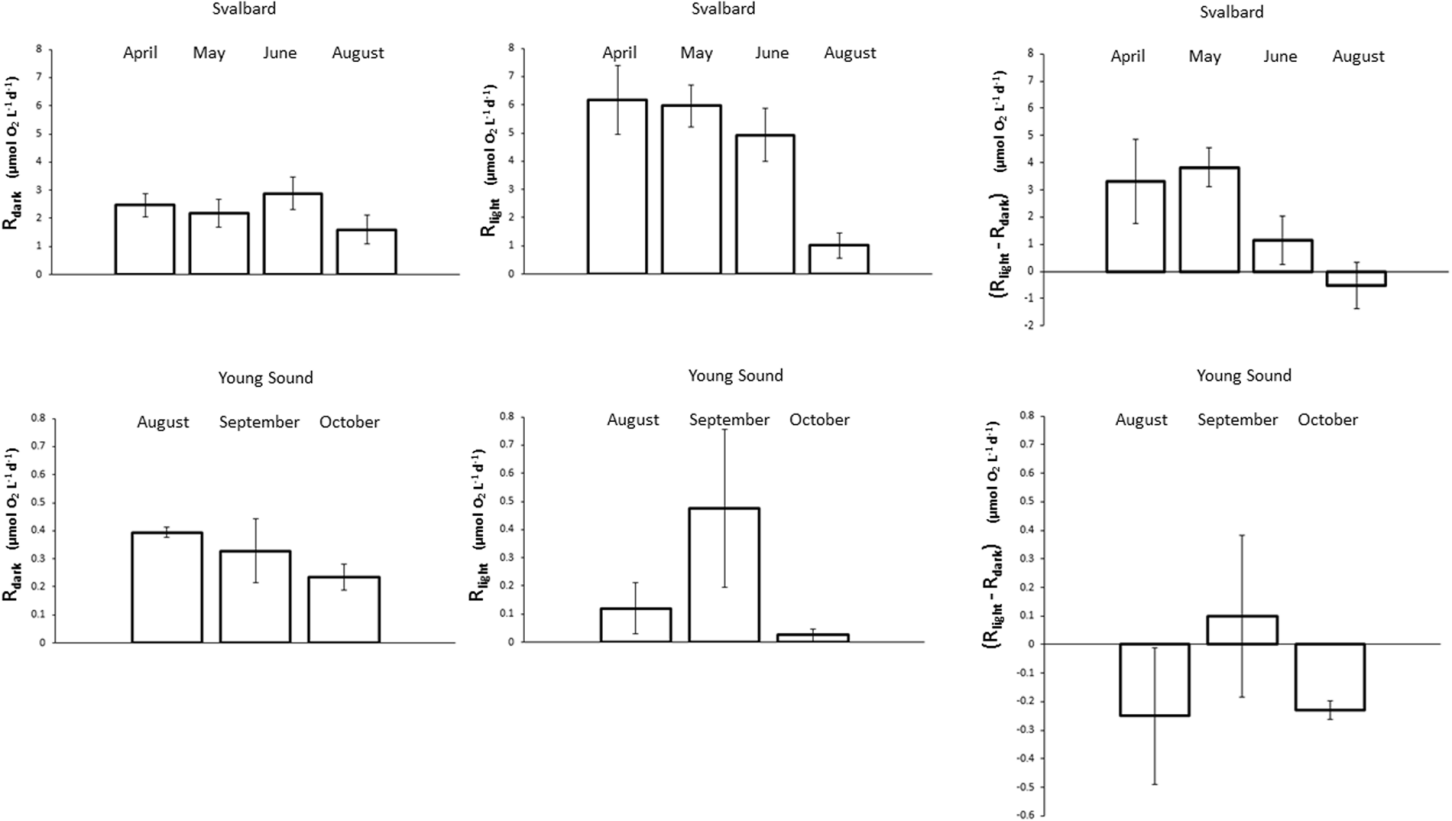
Mean (±SE) monthly respiration in the dark (R_dark_), respiration in the light (R_light_), and the difference between both (R_light_–R_dark_) in the Svalbard region and Young Sound.

Plankton community respiration varied three orders of magnitude among communities, and was significantly higher in the communities sampled in the Svalbard region compared to those sampled in Young Sound (Kruskal-Wallis test, P < 0.01), both when measured in the light and in the dark (Table 1). Mean monthly community respiration rates in the Svalbard region were highest in April and lowest in August (Fig. 1), although these differences were only significant for respiration rates measured in the light (Kruskal-Wallis test, P = 0.013), when rates measured in August were significantly lower than those measured in April and May (Dunn’s test, P < 0.05), but not June (Dunn’s test, P = 0.27). The statistical significance of seasonal differences could not be tested for the communities examined in Young Sound (Fig. 1), due to the limited number of estimates available and the fact that respiration rates in Young Sound were not evaluated in the spring, when the area is still fully covered by sea ice.

**Table 1:** Mean ± standard error (SE), median, range and number of estimates (N) for volumetric (µmol O_2_ L^-1^ d^-1^) rates (respiration in the dark and in the light), and for the ratio between both.

Svalbard															
	R_dark_					R_light_					R_light_:R_dark_				
	mean ± SE	median	min	max	N	mean ± SE	median	min	max	N	mean ± SE	median	min	max	N
Surface	2.50 ± 0.42	2.06	0.21	8.25	28	3.18 ± 0.64	2.68	0.09	9.56	20	2.87 ± 1.08	1.40	0.03	17.88	18
20% PAR	2.68 ± 0.72	1.66	0.12	17.69	25	6.67 ± 0.93	6.59	0.76	13.35	19	7.66 ± 3.31	2.06	0.57	52.51	18
DCM	2.40 ± 0.49	1.64	0.10	11.52	28	5.65 ± 0.92	4.01	0.44	15.80	27	5.56 ± 1.84	2.48	0.24	39.80	24
Overall	2.52 ± 0.31	1.76	0.10	17.69	81	5.20 ± 0.52	3.97	0.09	15.80	66	5.38 ± 1.26	1.85	0.03	52.51	60
**Young Sound**															
Surface	0.30 ± 0.09	0.25	0.03	0.89	9	0.51 ± 0.24	0.68	0.12	0.73	3	2.27 ± 2.09	0.82	0.32	5.67	3
20% PAR	—	—	—	—	—	—	—	—	—	—	—	—	—	—	—
DCM	0.34 ± 0.10	0.29	0.06	0.77	7	0.02 ± 0.01	0.02	0.01	0.04	3	0.12 ± 0.04	0.14	0.05	0.17	3
Overall	0.32 ± 0.06	0.27	0.03	0.89	16	0.27 ± 0.15	0.08	0.01	0.73	6	1.20 ± 0.99	0.25	0.05	5.67	6
**Overall**															
Surface	1.96 ± 0.35	1.22	0.03	8.25	37	2.83 ± 0.59	2.55	0.09	9.56	23	2.78 ± 0.94	1.37	0.03	17.88	21
20% PAR	2.68 ± 0.72	1.66	0.12	17.69	25	6.67 ± 0.93	6.59	0.76	13.35	19	7.66 ± 3.31	2.06	0.57	52.51	18
DCM	1.99 ± 0.41	1.26	0.06	11.52	35	5.09 ± 0.88	3.97	0.01	15.80	30	4.96 ± 1.66	1.18	0.05	39.80	27
Overall	2.16 ± 0.27	1.47	0.03	17.69	97	4.78 ± 0.50	3.62	0.01	15.80	72	5.00 ± 1.15	1.57	0.03	52.51	66

**Figure 2:**
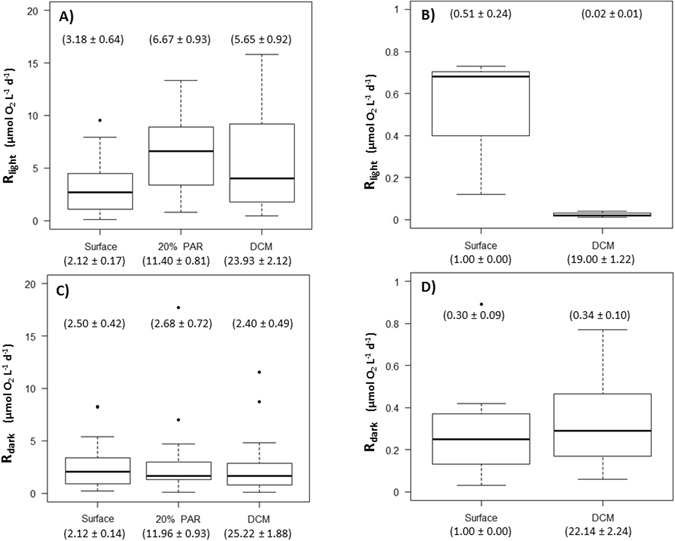
Box-and-Whisker plots showing the distribution of community respiration (**A**) in the light in Svalbard, (**B**) in the light in Young Sound, (**C**) in the dark in Svalbard and (**D**) in the dark in Young Sound, for the depths sampled (mean depth ± SE in parentheses). The boxes show the median rate plus the lower (25%) and upper (75%) quartiles, the whiskers indicate 1.5 times the interquartile range, and the points show outliers. Numbers above the boxes are the mean rates.

Community respiration rates in the light differed with depth (Kruskal-Wallis test, P = 0.0015, Fig. 2A,B), with the respiration rate in the light in communities sampled at the depth receiving 20% of PAR (photosynthetically active radiation) being significantly higher (Dunn’s test, P = 0.0014) than those sampled at the depth of chlorophyll maximum, DCM, and surface samples having the minimum mean respiration rate in the light among the three depths (Table 1). In contrast, community respiration in the dark did not differ with depth (Kruskal-Wallis test, P = 0.53), with comparable mean values across depths (Table 1 and Fig. 2C,D).

**Figure 3:**
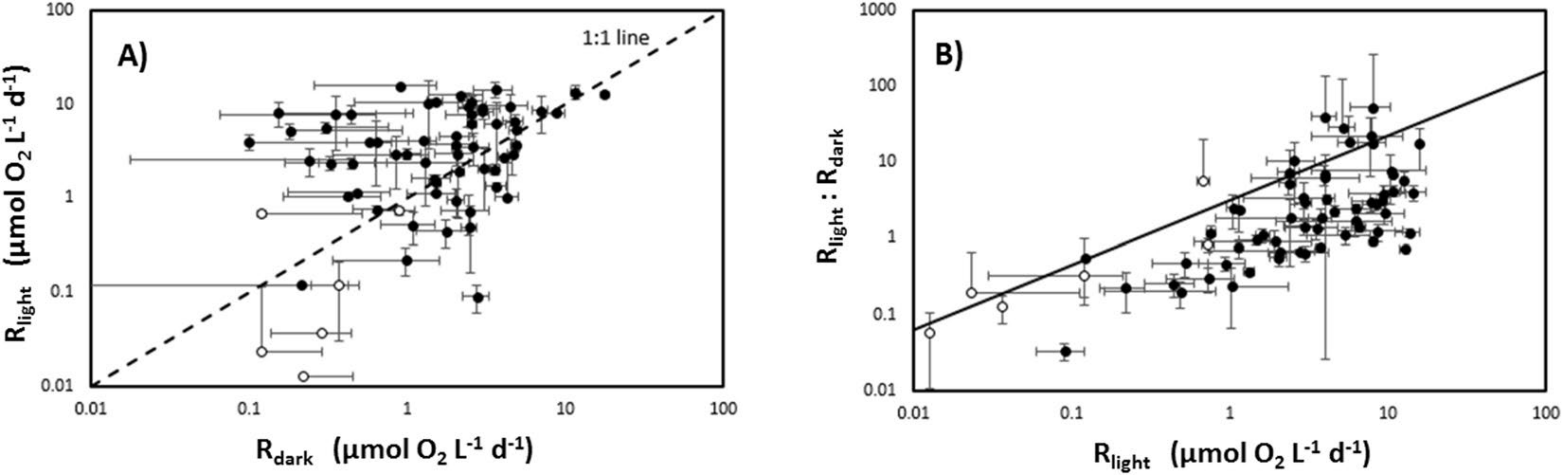
The relationship between (**A**) respiration in the dark (R_dark_) and that in the light (R_light_), and (**B**) respiration in the light (R_light_) and the ratio between respiration in the light and that in the dark (R_light_:R_dark_) in the Svalbard region (black symbols) and in Young Sound (white symbols). The solid line in (**B**) shows the fitted regression line Ln (R_light_:R_dark_) = −0.02 + 0.68 * Ln (R_light_) (µmol O_2_ L^−1^ d^−1^), R^2^ = 0.50, P < 0.001. Error bars are the SE.

Community respiration rates evaluated in the light and in the dark differed consistently (Wilcoxon signed rank test, p < 0.0001), with respiration rates in the light tending to be greater than those measured in the dark (Fig. 3A). The difference between R_light_ and R_dark_ did not differ significantly with depth (Kruskal-Wallis test, P = 0.19), but was greatest in May, when R_light_ tended to be much higher than R_dark_, compared to a smaller absolute difference in June and August (Fig. 1, Kruskal-Wallis test, P = 0.0085; Dunn’s test, P < 0.05). Closer examination showed that community respiration rates evaluated in the light and in the dark differed significantly for the communities evaluated in the Svalbard region (Wilcoxon signed rank test, p < 0.001), but not so for those in Young Sound (Wilcoxon signed rank test, p = 0.22), where community respiration rates were consistently low. The ratio R_light_:R_dark_ varied three orders-of-magnitude across communities (Table 1), and increased significantly (R^2^ = 0.50, P < 0.001) in communities showing high respiration in the light (Fig. 3B). The fitted regression equation showed that the ratio R_light_:R_dark_ was scaled to the 2/3 power of R_light_ (Fig. 3B), so that the R_light_ and R_dark_ were similar for R_light_ of the order of 1 µmol O_2_ L^−1^ d^−1^, but R_light_ was four-fold greater than R_dark_ for high R_light_ rates of the order of 10 µmol O_2_ L^−1^ d^−1^ (Fig. 3B).

**Figure 4:**
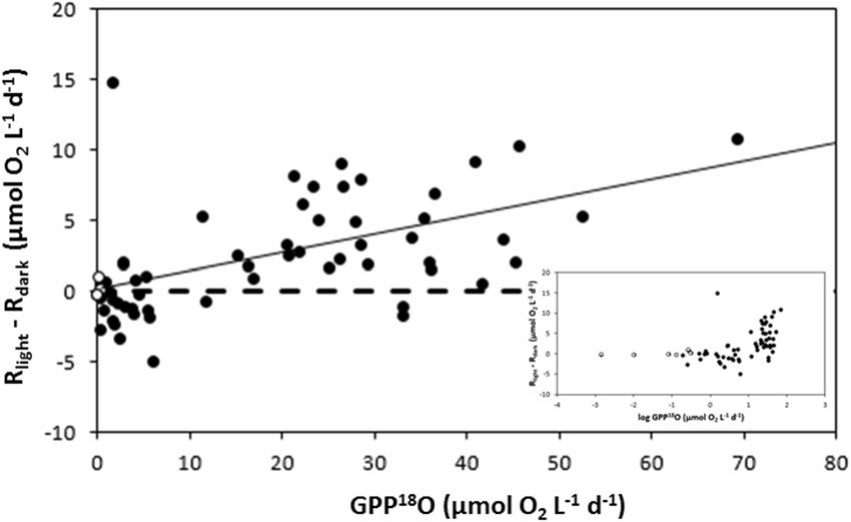
The relationship between gross primary production (GPP^18^O) and the difference between community respiration in the light and that in the dark (R_light_–R_dark_) in the Svalbard region (black symbols) and in Young Sound (white symbols). The insert shows the same figure with log- transformed gross primary production (log GPP^18^O), to allow examination of the values at low GPP^18^O values.

The difference between community respiration rates evaluated in the light and in the dark increased significantly with increasing GPP^18^O rates (Fig. 4), with no significant difference in community respiration rates at GPP^18^O rates <10 µmol O_2_ L^−1^ d^−1^ (Fig. 4).

## Discussion

The respiration of plankton communities is a major component of the carbon budget of the oceans^2^. Yet, estimates of community respiration rates are much less frequent than those of primary production, particularly in the Arctic Ocean where community respiration rates had thus far been evaluated only in the dark^11–15^. We found that R_light_ tended to be significantly higher than R_dark_ across the Arctic plankton communities tested. This is consistent with the majority of reports concluding that respiration in the light tends to be greater than that in the dark^6, 7, 16–18^, involving all, except two^19, 20^, published reports comparing such rates. However, the underestimation of respiration rates derived from measuring respiration rates in the dark may be particularly acute for Arctic plankton communities, which experience a 24-hour photoperiod during much of the year.

The estimates of R_light_ provided here represent the first assessment of respiration under ambient solar radiation for Arctic plankton communities. Previous comparison of R_light_ and R_dark_ for polar plankton communities derived from the Southern Ocean, where two studies had been conducted^6, 18^. These studies also concluded that respiration in the light tends to be greater than that in the dark. The mean vertically-integrated R_light_:R_dark_ ratio was reported to be 1.95 in a summer cruise around 76 °S in the Ross Sea^6^; and to range between 1.2 and 2 for spring and summer, respectively, in a transect from 52 to 70 °S across the Antarctic Polar Front region^18^. The median R_light_:R_dark_ ratio in our study was 1.57, within the range of values reported for Southern Ocean plankton communities^6, 18^. We found, however, that the R_light_:R_dark_ ratio increased as the 2/3 power of R_light_ so that the R_light_:R_dark_ increased from an average value of 1.37 at the median R_light_ measured here (3.62 µmol O_2_ L^−1^ d^−1^) to an average value of 17.56 at the highest R_light_ measured here (15.8 µmol O_2_ L^−1^ d^−1^).

**Figure 5:**
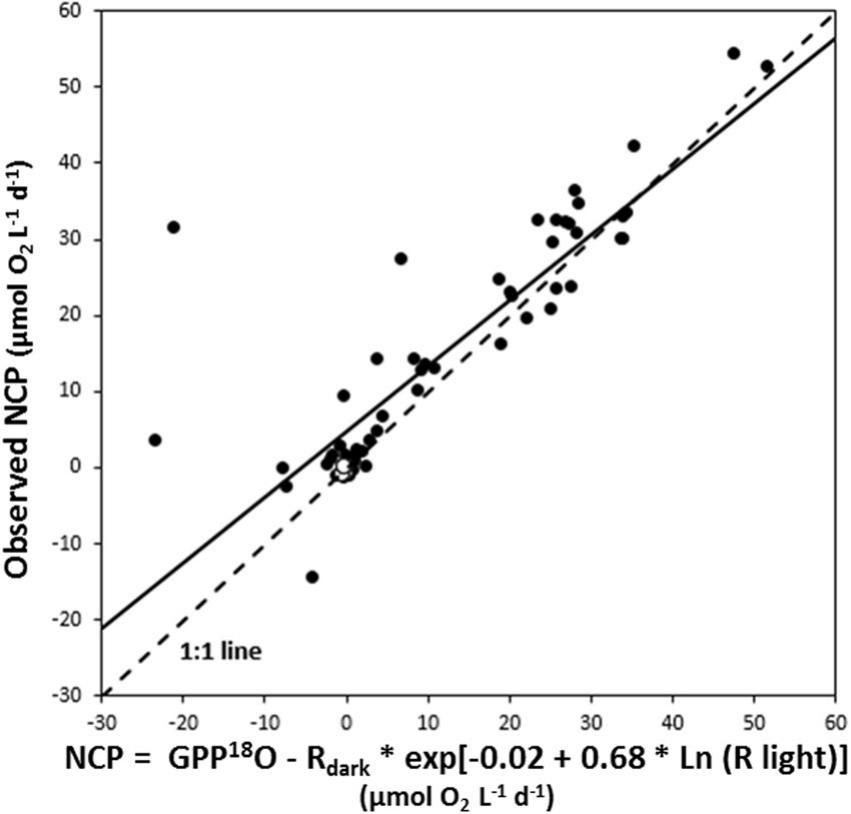
The relationship between NCP calculated as GPP^18^O - R_dark_ * exp[−0.02 + 0.68 * Ln (R light)] and observed net community production (NCP) in the Svalbard region (black symbols) and in Young Sound (white symbols). The solid line shows the fitted regression equation: y = 0.86 (±0.06) × +4.81 (±1.19) (R² = 0.74, p < 0.001, N = 66).

Estimates of gross primary production obtained directly using the ^18^O method tend to be greater than those calculated as the difference between NCP and R_dark_, which comprise all of the estimated gross primary production thus far available for the Arctic Ocean^11–15^. This was interpreted to indicate that R_light_ tends to be higher than R_dark_^21^, as confirmed by our results. Indeed, when the estimates of R_dark_ obtained here are corrected for the underestimation derived from estimating this rate in the dark by multiplying them by the R_ligth_:R_dark_ ratio predicted from the regression equation in Fig. 3, the NCP predicted as the difference between GPP^18^O and this corrected R estimate is strongly consistent with the observed NCP (Fig. 5). Hence, whereas reported NCP for plankton communities in the Arctic Ocean^11–15^ are robust, previous estimates of gross primary production and respiration rates are underestimates. The reason for this is that the assumption, rejected by our experimental results, that R_light_ equals R_dark_ is particularly inadequate for the high Arctic, where plankton communities do not experience darkness within the photic zone during the 24 h photoperiods in spring and summer.

It has been suggested that R_light_ rates are higher than those in the dark due to the contribution of autotrophic metabolic processes, such as photoenhanced mitochondrial respiration, chlororespiration, photorespiration and/or the Mehler reaction^22^. Autotrophic respiration has also been proposed to dominate community R during the pre-bloom and bloom phases of the seasonal cycle in the Southern Ocean^23^. These observations are consistent with the observation that the difference between R_light_ and R_dark_ estimates increased with increasing gross primary production (Fig. 4). Figure 4 also shows that for GPP^18^O < 10 µmol O_2_ L^−1^ d^−1^, most R_dark_ tend to be higher than R_light_, reflecting that metabolic processes supporting R_light_ may be specially enhanced over a GPP^18^O threshold of 10 µmol O_2_ L^−1^ d^−1^, below which dark processes prevail.

In conclusion, the results presented show that respiration in the light tends to be much higher than that in the dark in productive communities, whereas both values are low in communities with low productivity. Periods of high production, particularly the spring bloom, contribute disproportionately to the annual metabolic budget of the Arctic Ocean^11^. Estimates of net community production in the Arctic^11, 15^, which are derived from incubations in the light, are not affected by the bias introduced by dark incubations to estimate respiration rates. However, these procedures would have led to underestimate the gross primary production of Arctic communities in the summer, where this was derived as the difference between NCP and respiration rates.

## Methods

**Figure 6:**
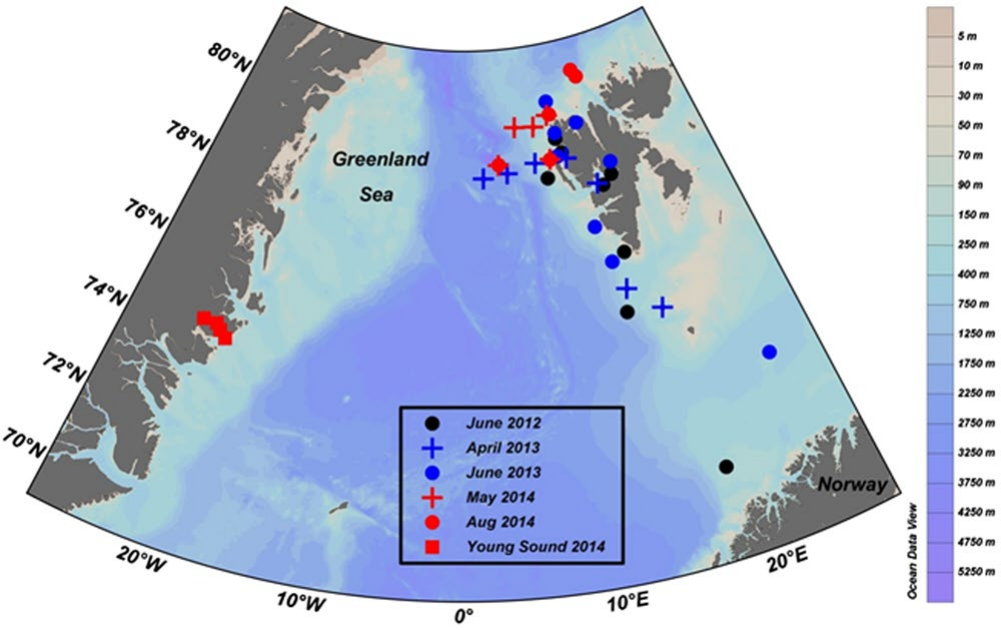
Location of the stations sampled. Map created with Ocean Data View software (version 4.6.3, http://odv.awi.de/).

Plankton community respiration (R) in the dark and under ambient irradiance conditions was evaluated in both sides of the Greenland Sea, the western margin of Svalbard region and Young Sound fjord, in NE Greenland (Fig. 6). R was evaluated in five cruises in Svalbard, in 2012 (from 9 to 16 June), 2013 (27 April to 4 May; and 6 to 14 June) and 2014 (16 to 27 May; 8 to 14 August). Four stations were sampled in Young Sound in each of August, September and October 2014.

In the cruises conducted in the Svalbard region, water samples were collected using a Rosette sampler system fitted with 5 L Niskin bottles and a calibrated CTD, at three different depths: surface (2.12 ± 0.13 m), DCM (24.56 ± 1.63 m), which receives, on average, 1% of the incident irradiance, and at an intermediate depth (13.56 ± 0.93 m) between surface and DCM, receiving 20% of the incident radiation on the surface. Only two depths (surface and DCM) were sampled in Young Sound, where temperature and salinity were collected from a CTD cast, and water samples were collected with 5 L Niskin bottles.

Plankton community respiration rates were estimated using two methods: (1) respiration in the dark (R_dark_) was assessed by evaluating oxygen consumption after incubation of samples in the dark, by high-precission Winkler titration^24, 25^ in Svalbard cruises and by visual end-point detection^26^ in Young Sound, and (2) respiration in the light (R_light_) was assessed as the difference between gross primary production (GPP^18^O), evaluated using H_2_^18^O additions^27^, and net community production (NCP), evaluated from oxygen changes resolved using high-precision Winkler titration^24, 25^ in Svalbard and using visual end-point detection^26^ in Young Sound, of samples incubated under the incident solar radiation. Daily R_light_ rates were corrected for those communities that were exposed to less than 24 hours of light (only five communities in September and October in Young Sound). The rates determined based on disolved oxygen changes in Young Sound, 12 out of 147 respiration rates reported here, carry considerable error, as the titration end point was determined visually, as a titrator was not available. The precision obtained (expressed as SD of average in %) for O_2_ concentration measurements with this procedure was 0.15%.

Per each depth, a set of seven replicated 100-mL narrow-mouth Winkler bottles was fixed immediately to evaluate the initial oxygen content, and two sets were incubated under light and dark conditions for 24 hours. Incubations were done in water baths on deck (maintained at the *in situ* temperature of the surface water, ±1 °C, through continuous water flow from the surface) in Svalbard; and *in situ* in Young Sound. Neutral screens were used to reduce incident irradiance as to mimic the light environment *in situ*. Dissolved oxygen concentrations were determined by automated high-precision Winkler titration with a potentiometric end-point Metrohm 808 Titrando^28^ in the Svalbard communities and using starch as indicator for end-point detection in the Young Sound communities. R_dark_ and NCP were calculated from changes in dissolved oxygen concentrations, before and after incubation of samples under “dark” and “light” conditions, respectively, for 24 h in Svalbard and 48 h in Young Sound. As a consequence of the low rates and low precision of dissolved oxygen determination in Young Sound, the communities were incubated for 48 h, thereby experiencing changes that could be resolved with the techniques used there. On the other hand, long incubations may increase the risk of artifacts derived from bottle effects. Rates are reported in µmol O_2_ L^−1^ d^−1^ and standard errors were calculated using error propagation. In order to compare the R_light_:R_dark_ ratios obtained here with those observed in the past, we surveyed the literature for results reported in the past^6, 7, 16–18, 27^. An extreme value reported by one of the studies^7^ (ratio R_light_:R_dark_ = 19), 8-fold higher than the rest, was excluded from the comparison.

For evaluation of GPP^18^O, eight 12-ml glass vials were filled per depth. Four replicate samples were immediately fixed (biological activity stopped) with 80 µl of saturated HgCl_2_ solution for later analysis of initial δ^18^O(O_2_) values, and stored upside down in darkness. The other four vials, containing beads inside to ensure mixing, were spiked with 80 µl and 200 µl of 98% H_2_^18^O in Svalbard and Young Sound communities, respectively. After being closed, these spiked vials were immediately agitated, to ensure that H_2_^18^O was homogeneously distributed inside the vial. The spiked samples were incubated together with the Winkler bottles under “light” conditions. After the 24-hour incubation, vials were fixed with 80 µl of saturated HgCl_2_ solution and stored upside down in darkness.

At the stable isotope laboratory, a 4-ml headspace was generated in each vial, by flushing with a helium flow. The vials were left for equilibration at room temperature for 24 hours. The δ^18^O of dissolved oxygen in the headspace was measured in a Finnigan GasBench II attached to a Finnigan DeltaPlusXP isotope ratio mass spectrometer, with precision better than 0.1‰. δ^18^O-H_2_O of spiked samples was measured in a liquid water isotope analyzer (Los Gatos Research), with precision of 0.1‰, and GPP^18^O was calculated^22^.

Statistical analysis were based on non-parametric tests (Wilcoxon signed rank test, Kruskal-Wallis test and Dunn’s test), as the data were skewed and not normally distributed, or log-transformed to homogenize the variance prior to fitting least squares linear regression equations.

## References

[1] Duarte, C. M., Agustí, S. & Regaudie-de-Gioux, A. In *The Role of Marine Biota in the Functioning of the Biosphere* (ed. Duarte, C. M.) 39–54 (Fundación BBVA, Bilbao, 2011).

[2] Del Giorgio PA, Duarte CM (2002). Respiration in the open ocean. Nature.

[3] Balkanski Y, Monfray P, Batle M, Heimann M (1999). Ocean primary production derived from satellite data: An evaluation with atmospheric oxygen measurements. Glob. Biogeochem. Cycles.

[4] Williams, P. J. le B. & Del Giorgio, P. A. In *Respiration in aquatic* ecosystems (eds Del Giorgio, P. A. & Williams, P. J. le B.) 1–18 (Oxford University Press, Oxford, 2005).

[5] Harris GP, Lott JNA (1973). Light intensity and photosynthetic rates in phytoplankton. J. Fish. Res. Board Can.

[6] Bender ML, Dickson ML, Orchardo J (2000). Net and gross production in the Ross Sea as determined by incubation experiments and dissolved O_2_ studies. Deep Sea Res. II.

[7] Robinson C (2009). Comparison of *in vitro* and *in situ* plankton production determinations. Aquat. Microb. Ecol..

[8] Vaquer-Sunyer R, Duarte CM, Wassmann P, Santiago R, Reigstad M (2010). Experimental evaluation of planktonic respiration response to warming in the European Arctic Sector. Polar. Biol..

[9] Holding JM (2013). Experimentally determined temperature thresholds for Arctic plankton community metabolism. Biogeosciences.

[10] Duarte CM (2012). Tipping elements in the Arctic marine ecosystem. Ambio.

[11] Vaquer-Sunyer R (2013). Seasonal patterns in Arctic planktonic metabolism (Fram Strait - Svalbard region). Biogeosciences.

[12] Regaudie-de-Gioux A, Duarte CM (2010). Plankton metabolism in the Greenland Sea during the polar summer of 2007. Polar Biol..

[13] Cottrell MT, Malmstrom RR, Hill V, Parker AE, Kirchman DL (2006). The metabolic balance between autotrophy and heterotrophy in the western Arctic Ocean. Deep-Sea Res. I.

[14] Sherr BF, Sherr EB (2003). Community respiration/production and bacterial activity in the upper water column of the central Arctic Ocean. Deep-Sea Res. I.

[15] Sejr MK (2014). Seasonal dynamics of autotrophic and heterotrophic plankton metabolism and pCO_2_ in a subarctic Greenland fjord. Limnol. Oceanogr..

[16] Dickson ML, Orchardo J (2001). Oxygen production and respiration in the Antarctic Polar Front region during the austral spring and summer. Deep Sea Res. II.

[17] Grande KD (1989). Primary production in the North Pacific gyre: a comparison of rates determined by the ^14^C, O_2_ concentration and ^18^O methods. Deep-Sea Res.

[18] Hitchcock GL, Vargo GA, Dickson ML (2000). Plankton community composition, production, and respiration in relation to dissolved inorganic carbon on the West Florida Shelf, April 1996. J. Geophys. Res..

[19] Marra J, Barber RT (2004). Phytoplankton and heterotrophic respiration in the surface layer of the ocean. Geophys. Res. Lett..

[20] González N, Gattuso JP, Middelburg JJ (2008). Oxygen production and carbon fixation in oligotrophic coastal bays and the relationship with gross and net primary production. Aquat. Microb. Ecol..

[21] Regaudie-de-Gioux A, Lasternas S, Agustí S, Duarte CM (2014). Comparing marine primary production estimates through different methods and development of conversion equations. Front. Mar. Sci.

[22] Bender ML, Orchardo J, Dickson ML, Barber R, Lindley S (1999). *In vitro* O_2_ fluxes compared with ^14^C production and other rate terms during the JGOFS Equatorial Pacific experiment. Deep Sea Res. I.

[23] Goldman, J. A. *et al*. Gross and net production during the spring bloom along the Western Antarctic Peninsula. *New Phytologist* **205**, 182–191 (2015). 28. Carpenter, J. H. The accuracy of the Winkler method for dissolved oxygen analysis. *Limnol. Oceanogr*. **10**, 135–140 (1965). 10.1111/nph.13125 25382393

[24] Carpenter JH (1965). The accuracy of the Winkler method for dissolved oxygen analysis. Limnol. Oceanogr..

[25] Carrit DE, Carpenter JH (1966). Comparison and evaluation of currently employed modifications of the Winkler method for determining dissolved oxygen in sea-water. J. Mar. Res..

[26] Grasshoff, K. Determination of oxygen. In *Methods of Seawater**Analysis* (ed. Grasshoff, K., Ehrhardt, M. & Kremling, K.) 61–72 (Verlag Chemie, Weinheim, 1983).

[27] Bender ML (1987). A comparison of four methods for determining planktonic community production. Limnol. Oceanogr.

[28] Oudot C, Gerard R, Morin P, Gningue I (1988). Precise shipboard determination of dissolved-oxygen (Winkler Procedure) for productivity studies with a commercial system. Limnol. Oceanogr.

